# Challenges of Investigating Community Outbreaks of Cyclosporiasis, British Columbia, Canada

**DOI:** 10.3201/eid1508.081585

**Published:** 2009-08

**Authors:** Lena Shah, Laura MacDougall, Andrea Ellis, Corinne Ong, Sion Shyng, Linda LeBlanc, the British Columbia Cyclospora Investigation Team

**Affiliations:** Public Health Agency of Canada, Ottawa, Ontario, Canada (L. Shah); British Columbia Centre for Disease Control, Vancouver, British Columbia, Canada (L. Shah, L. MacDougall, C. Ong, S. Shyng); Public Health Agency of Canada, Guelph, Ontario, Canada (A. Ellis); Canadian Food Inspection Agency, Ottawa (L. LeBlanc)

**Keywords:** Cyclospora, enteric infections, parasites, foodborne diseases, disease outbreaks, Canada, dispatch

## Abstract

Investigations of community outbreaks of cyclosporiasis are challenged by case-patients’ poor recall of exposure resulting from lags in detection and the stealthy nature of food vehicles. We combined multiple techniques, including early consultation with food regulators, traceback of suspected items, and grocery store loyalty card records, to identify a single vehicle for a cyclosporiasis outbreak in British Columbia, Canada, in 2007.

*Cyclospora cayetanensis* is an emerging coccidian parasite that causes outbreaks of protracted and relapsing gastroenteritis (*1*,*2*). Delays in clinical diagnosis caused by the waxing and waning symptoms of *Cyclospora* infection, coupled with a long incubation period (median 7 days) and concealed food vehicles (e.g., herbs), result in poor recall of food exposures. Therefore, outbreaks in which no common meal is eaten are even more difficult to solve. In 2007, the British Columbia Centre for Disease Control used early collaboration with the Canadian Food Inspection Agency (CFIA), grocery card shopping records, and product traceback for several suspected items simultaneously to successfully implicate a vehicle in a community *Cyclospora* outbreak.

## The Study

**Figure 1:**
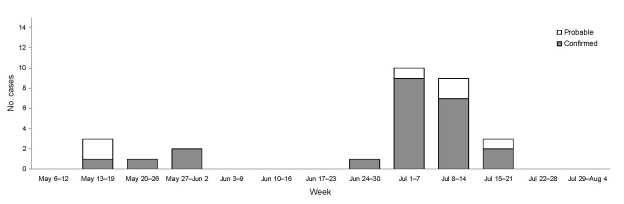
Confirmed and probable cases of cyclosporiasis (N = 29), by date of onset, British Columbia, Canada, May–August 2007.

From May 1 through July 30, 2007, a total of 29 cases of locally acquired *Cyclospora* infection were reported in British Columbia (Figure 1; Table 1). An initial investigation was conducted around the 6 laboratory-confirmed case-patients reported in the last 2 weeks of May and the first week of June (phase 1). No common exposure was reported, and case reports subsided. During the last week of June, case reports resumed, and phase 2 of the investigation was initiated. A total of 19 confirmed and 4 probable cases were identified with symptom onsets during June 28–July 20, 2008. Fifty-three percent of these cases occurred in male patients. No case-patients were hospitalized. Average time from symptom onset to positive laboratory result was 17 days (range 6–31 days).

**Table 1:** Cyclosporiasis case definitions, British Columbia, Canada, May 1–July 31, 2007

Case type	Definition
Confirmed	British Columbia residents who had not traveled outside Canada and the United States within 2 weeks before symptom onset and in whom *Cyclospora* species oocysts* were detected by microscopy on or after May 1, 2007
Probable	Clinical illness compatible with *Cyclospora* infection (i.e., watery diarrhea and/or bloating, cramps, loss of appetite, loss of weight), no travel outside Canada and the United States within 2 weeks before symptom onset, and onset of symptoms within a week of a person with whom they shared food exposures and who had a laboratory-confirmed case

*Laboratory-confirmed cases were reported to public health by medical diagnostic laboratories and specimens forwarded for confirmation to the public health reference laboratory. The morphologic identification of *Cyclospora* spp. oocysts included shape (spherical), size (8–10 μm in diameter), oocyst wall (well-defined), internal contents with refractile globules, autofluorescence, and modified acid-fast or safranin staining (*1*).

During phase 2, a total of 17 confirmed case-patients were interviewed with hypothesis-generating questionnaires about items eaten in the 2 weeks before symptom onset. The instrument included questions about restaurant history with meal details; grocery stores frequented; and yes/no questions about >70 fruits and vegetables, 8 herbs, and 16 mixed foods (e.g., salsa, pesto) previously implicated in outbreaks of foodborne disease. No common restaurants or events were identified.

Frequently reported foods were compared with population controls from Canadian (Waterloo, Ontario) and American (Oregon; US Foodborne Diseases Active Surveillance Network [FoodNet]) published food consumption surveys (*3*–*5*). Although such measurements may be limited by the timing of questionnaire administration and the recall period considered, they can be useful comparators during the hypothesis-generating stages of an investigation. By the end of phase 2, strawberries, cilantro, and sweet basil were reported more often than expected by case-patients (Table 2). Garlic and red peppers also were commonly eaten by case-patients; however, population comparisons were unavailable. Eighty-eight percent of case-patients reported having eaten romaine lettuce; 85% of controls in the Waterloo survey (*4*,*5*) had eaten lettuce of any type, and romaine lettuce consumption was much less commonly reported in the FoodNet survey (*3*) (Table 2). Other foods assessed were not eaten more often than expected. A formal case–control study was considered premature in the early stages of phase 2 because no strong hypothesis emerged from early interviews and comparisons to population controls. We further explored the plausibility of various hypotheses through a combination of methods described below that allowed room for additional hypotheses to emerge or existing hypotheses to strengthen as cases accrued.

**Table 2:** Food items eaten more often by case-patients than by population controls, British Columbia, Canada, May 1–July 31, 2007*

Food item	No. (%) case-patients	Controls, %	
		Waterloo survey	US FoodNet
Strawberries	16–17 (94–100) (1 unsure)	32	28
Romaine lettuce	15 (88)	Any lettuce, 84	37
Garlic	12 (71)	NA	NA
Red peppers	14 (82)	NA	NA
Cilantro	12–15 (70–88) (3 unsure)	8	NA
Basil	14–16 (82–94) (2 unsure)	12	NA

*Case-patients were 17 persons with laboratory-confirmed cases interviewed during phase 2 (June 24–July 21, 2007). US FoodNet, Foodborne Diseases Active Surveillance Network; NA, not applicable.

Detailed questionnaires asked whether foods were eaten in a restaurant or were store-bought and about type of packaging and method of preparation (because *C*. *cayetanensis* is heat-sensitive) (*6*). We reinterviewed early case-patients using the second questionnaire and interviewed later case-patients using both questionnaires. In phase 1, garlic eaten at restaurants by all 4 persons with confirmed infections were traced back to different suppliers; only 1 case-patient ate raw garlic in a restaurant. Three case-patients also reported eating cooked garlic at home; cooking would have inactivated the pathogen.

Early and proactive collaboration with CFIA involved a general assessment of the country of origin and distribution patterns for frequently eaten foods. According to CFIA records, romaine lettuce and red peppers sold during the exposure period were not imported from a known *Cyclospora*-endemic country and were widely distributed in Canada and the United States. This was not consistent with case distribution (R. Cardinal, CFIA, pers. comm.).

**Figure 2:**
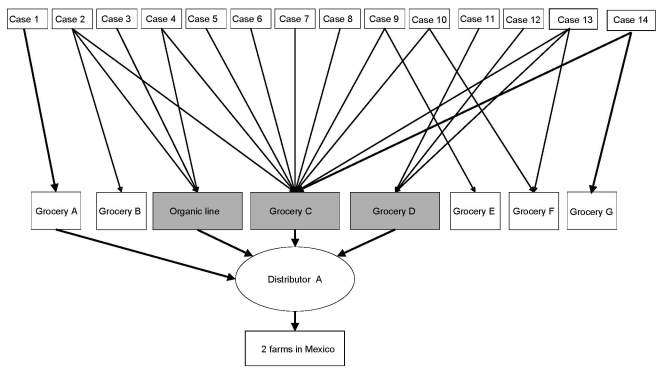
Traceback of basil eaten by persons with confirmed cyclosporiasis (N = 14), British Columbia, Canada, May–August 2007.

Because interviews, population control comparisons, and product distribution limited suspected foods to strawberries, cilantro, and basil, we began preliminary traceback of all 3 suspected items. Environmental health officers and regional CFIA staff interviewed grocery store owners, restaurant managers, and distributors to trace produce to its supplier. Local strawberries eaten by case-patients from 3 small markets were traced back to 2 local farms in geographically separate regions of British Columbia. Cilantro eaten by case-patients was traced to 2 suppliers; both supplied home-grown rather than imported produce. Of 14 case-patients with confirmed basil exposures, 4 (57%) ate only organic basil supplied by distributor A. Additionally, 4 (29%) reported multiple basil exposures, including exposure to organic basil from distributor A (Figure 2). In British Columbia, organic basil enjoys a smaller market share than the conventional product.

In phase 2, 12 (71%) of 17 case-patients reported shopping at grocery C. Records of any grocery store purchases for the households of 8 consenting case-patients were obtained through grocery C’s savings card program; other case-patients were not cardholders. All purchase histories were requested for 1 month before symptom onset to account for the typical incubation period plus product shelf life. Records from 3 (38%) case-patients showed purchases of the same organic basil supplied by distributor A. Two case-patients had bought organic basil on the same day at the same location. Of the remaining 5 case-patients who recalled purchasing organic basil but whose consumer card records did not confirm it, 2 had not used their cards for large portions of the incubation period.

We collected supplier information for organic basil during a visit to the distribution warehouse and local farm site of distributor A. The remaining 2 (14%) case-patients with basil exposure previously unlinked to distributor A were confirmed through trace-forward from distributor A. The first had eaten organic basil at a smaller market supplied by distributor A under another trade name. The second had eaten conventional basil from a grocery store supplied by distributor A. Distributor A confirmed using organic basil to supplement conventional basil shipments when supply was low. Late summer outbreaks of cyclosporiasis in British Columbia are unusual; distributor A confirmed that imported product was used throughout the summer in 2007 because of a poor local growing season.

All case-patients in phase 2 who recalled basil exposure (82%) could have been exposed to organic basil from distributor A. Once this common vehicle was identified, CFIA conducted a full traceback of organic basil by using formal documentation including invoices, shipment numbers, and airway bills. The suspected imported basil was no longer available for testing. Using distributor A invoices, we identified a specific shipment of organic basil imported from 1 of 2 Mexican supplier farms, and CFIA notified Mexican authorities. The Mexican farm was located in a region previously linked to cyclosporiasis outbreaks (R. Cardinal, CFIA, pers. comm.).

## Conclusions

Detailed interviews, modified traceback of several suspected items, and information about product distribution and market share led to organic basil as a primary hypothesis. Food regulators could pinpoint a specific shipment and trace it to its origin because consumer cards provided the exact purchase dates for basil that case-patients could not recall. Overall, the approach used in this investigation increased the work load typically requested of team members during foodborne outbreaks. However, this combination of investigative methods successfully identified a single vehicle during a community cyclosporiasis outbreak where a common menu was not available.
